# Reply: When Discrimination Transports but the Operating Point Does Not: AI-ECG for Left Ventricular Dysfunction

**DOI:** 10.1016/j.jacadv.2026.103136

**Published:** 2026-08-13

**Authors:** Hak Seung Lee, Joon-myoung Kwon, Sora Kang, Tae-Min Rhee, Heesun Lee

**Affiliations:** a Digital Healthcare Institute, Sejong Medical Research Institute, Bucheon, Republic of Korea; b Medical AI Co., Ltd., Seoul, Republic of Korea; c Department of Internal Medicine, Seoul National University College of Medicine, Seoul, Republic of Korea; d Division of Cardiology, Seoul National University Hospital Healthcare System Gangnam Centre, Seoul, Republic of Korea

We thank Dr Lin and colleagues for their thoughtful letter. They urge a clear separation of discrimination, operating point, and calibration, and we agree that this distinction is central to the safe deployment of artificial intelligence–enabled electrocardiogram (AI-ECG).

First, on calibration. We agree that predicted-probability calibration should be reported whenever AI-ECG outputs are used to support risk-based decisions beyond binary triage.^1^^,^^2^ Our study was designed primarily to evaluate discrimination and rule-out performance in an asymptomatic screening population; with only 32 cases of left ventricular systolic dysfunction (left ventricular ejection fraction ≤40%; 0.054% of the cohort), calibration-in-the-large and, in particular, calibration slope would be estimated too imprecisely to be informative in this cohort. Such metrics are better assessed in higher-prevalence deployment settings.

Second, the 3 properties answer different questions. Discrimination transported from our development cohort (area under the curve: 0.973) to Kenya (0.96).^1^^,^^2^ The operating point did not—but, as our translational outlook anticipated, this is addressable by setting the threshold on local data rather than importing the development cutoff of 9.7. The authors' third recommendation aligns with this.

Third, although our cohort could not support precise calibration estimates, evidence for this algorithm family is not absent. A recent independent, head-to-head validation against cardiac magnetic resonance evaluated a related version of this model and found it slightly underestimated observed risk, with only minor changes in sensitivity and specificity after recalibration.^3^ It suggests that calibration error, when present, may be detectable and potentially correctable through local recalibration; however, it should not be taken as direct calibration evidence for the exact locked model used in either deployment.

Fourth, on false positives, we share the authors' reading. As they note, Kenyan false positives had higher left ventricular mass index, higher E/e′, and more prior infarction and atrial fibrillation; in our cohort, only 16.4% were entirely normal. Recent work indicates that AI-ECG models for left ventricular systolic dysfunction behave less like single-condition classifiers and more like broad markers of cardiovascular pathology.^4^ Viewed this way, some loss of specificity in higher-risk or more comorbid deployment populations may reflect clinically meaningful cardiac abnormality rather than random model error, limiting what threshold adjustment alone can recover. Accordingly, we further agree with the authors that specificity and calibration are best reported across risk strata rather than in aggregate.

Fifth, the value of our study lies in rule-out within an asymptomatic, general-population setting. At a prevalence of 0.054%, the intended clinical utility was high-sensitivity rule-out and triage, with a very high negative predictive value in that screening context rather than probability-based diagnosis. The Kenyan study is an excellent investigation in a resource-limited setting and warrants a somewhat different analytic lens.

Finally, a fair evaluation methodology for these new AI-ECG models is not yet established.^5^ We need standardized frameworks that move beyond discrimination alone to encompass calibration and operating-point transportability across deployment populations, and we welcome this discussion alongside the authors.
